# Patient Acceptable Symptom State Thresholds for IKDC-SKF and KOOS at the 10-Year Follow-up After Anterior Cruciate Ligament Injury: A Study From the Delaware-Oslo ACL Cohort

**DOI:** 10.1177/23259671241250025

**Published:** 2024-05-29

**Authors:** Anouk P. Urhausen, Hege Grindem, Lina H. Ingelsrud, Ewa M. Roos, Karin Grävare Silbernagel, Lynn Snyder-Mackler, May Arna Risberg

**Affiliations:** † Department of Sports Medicine, Norwegian School of Sport Sciences, Oslo, Norway; ‡ Oslo Sports Trauma Research Center, Department of Sports Medicine, Norwegian School of Sport Sciences, Oslo, Norway; § Department of Orthopaedic Surgery, Copenhagen University Hospital Hvidovre, Copenhagen, Denmark; ‖ Center for Muscle and Joint Health, Department of Sports Science and Clinical Biomechanics, University of Southern Denmark, Odense, Denmark; ¶ Department of Physical Therapy, University of Delaware, Newark, Delaware, USA; # Division of Orthopedic Surgery, Oslo University Hospital, Oslo, Norway; Investigation performed at the Department of Sports Medicine, Norwegian School of Sport Sciences, Oslo, Norway, and the Department of Physical Therapy, University of Delaware, Newark, Delaware, USA

**Keywords:** anterior cruciate ligament, interpretability, Patient Acceptable Symptom State, patient-reported outcomes, validity

## Abstract

**Background:**

Clinicians need thresholds for the Patient Acceptable Symptom State (PASS) and Treatment Failure to interpret group-based patient-reported outcome measures after anterior cruciate ligament (ACL) injury. Validated thresholds that are crucial for accurately discerning patient symptom state and facilitating effective interpretation have not been determined for long-term follow-up after ACL injury.

**Purpose:**

To calculate and validate thresholds for PASS and Treatment Failure for the International Knee Documentation Committee Subjective Knee Form (IKDC-SKF) and the Knee injury and Osteoarthritis Outcome Score (KOOS) subscales at the 10-year follow-up after ACL injury.

**Study Design:**

Cohort study; Level of evidence, 3.

**Methods:**

A total of 163 participants with unilateral ACL injury (treated with reconstruction or rehabilitation alone) from the Delaware-Oslo ACL Cohort were included. Thresholds for PASS were calculated for IKDC-SKF and KOOS subscales using anchor-based predictive modeling and receiver operating characteristic (ROC) analysis. Too few participants had self-reported Treatment Failure to calculate thresholds for that outcome. Nonparametric bootstrapping was used to derive 95% CIs. The criterion validity of the predictive modeling and ROC-derived thresholds were assessed by comparing actual patient-reported PASS outcome with the calculated PASS outcome for each method of calculation and calculating their positive and negative predictive values with respect to the anchor questions.

**Results:**

A total of 127 (78%) participants reported satisfactory symptom state. Predictive modeling PASS thresholds (95% CIs) were 76.2 points (72.1-79.4 points) for IKDC-SKF, 85.4 points (80.9-89.2 points) for KOOS Pain, 76.5 points (67.8-84.7 points) for KOOS Symptoms, 93.8 points (90.1-96.9 points) for KOOS activities of daily living, 71.6 points (63.4-77.7 points) for KOOS Sports, and 59.0 points (53.7-63.9 points) for KOOS quality of life (QoL). Predictive modeling thresholds classified 81% to 93% of the participants as having satisfactory symptom state, whereas ROC-derived thresholds classified >50% as unsatisfied. The thresholds for IKDC-SKF, KOOS Sports, and KOOS QoL resulted in the most accurate percentages of PASS among all identified thresholds and therefore demonstrate the highest validity.

**Conclusion:**

Predictive modeling provided valid PASS thresholds for IKDC-SKF and KOOS at the 10-year follow-up after ACL injury. The thresholds for IKDC-SKF, KOOS Sports, and KOOS QoL should be used when determining satisfactory outcomes. ROC-derived thresholds result in substantial misclassification rates of the participants who reported satisfactory symptom state.

Patient-reported outcome measures (PROMs) are commonly used to evaluate patient-perceived knee symptoms and function after anterior cruciate ligament (ACL) injury.^9,52,53^ However, clinicians and policymakers struggle to interpret the outcomes of PROMs on scales from 0 to 100 – what is regarded as a satisfactory outcome for a patient?^40^ Knowing whether patients are satisfied with their current knee status is of greater clinical significance than understanding the value of a PROM.^5,30^

Converting a PROM value into a meaningful measure of satisfactory outcomes helps to better interpret outcomes of PROMs at a group level, as in national ACL registries, large cohorts, and clinical trials.^4,7,37,46,51^ Such measures can inform clinicians and guide patient counseling by setting realistic expectations. Hence, the Patient Acceptable Symptom State (PASS) and Treatment Failure were developed.^24^ PASS is a simple and clinically relevant measure to judge a symptom state that the patient describes as satisfactory.^11,47^ In other words, PASS refers to a state beyond which patients can be expected to feel good at a given timepoint.^40^ Treatment Failure identifies patients who find their treatment has failed them. Thresholds reflecting PASS or Treatment Failure can be calculated and used for a given PROM and population to identify what constitutes a satisfactory or failed outcome for patients. Currently, valid thresholds are lacking to interpret the long-term outcomes of commonly used PROMs after ACL injury.^30^

Ensuring the validity of thresholds is crucial to accurately discern patient knee status and facilitate effective interpretation of the PROMs.^32^ Various methods are used to derive thresholds.^14,16,29^ PASS and Treatment Failure thresholds are best calculated by linking the PROM score to an anchor question,^47^ with receiver operating characteristic (ROC) analysis being traditionally used and predictive modeling being recently recommended as they provide more accurate thresholds.^43,45^ Nevertheless, we do not know how accurately these thresholds reflect patient perception of satisfactory symptom state.

Accordingly, we aimed to calculate PASS and Treatment Failure thresholds for long-term outcomes after an ACL injury and to validate their performance in identifying the satisfactory symptom state or failed treatment. We hypothesized that predictive modeling would yield more accurate PASS thresholds than ROC analysis.

## Methods

This was a prospective cohort study from the prospective Delaware-Oslo ACL Cohort. The outcomes of interest were the International Knee Documentation Committee Subjective Knee Form (IKDC-SKF) and the Knee injury and Osteoarthritis Outcome Score (KOOS) questionnaires,^6,26,27,34,41,42^ as well as single-item questions on PASS and Treatment Failure.^24^ The University of Delaware-Oslo ACL and the Regional Ethical Committee for South-Eastern Norway approved this study, and all participants provided written informed consent before data collection.

### Participants and ACL Treatment Algorithm

A total of 300 participants were enrolled between 2006 and 2012 in the University of Delaware, Newark, Delaware, United States, and the Norwegian Sports Medicine Clinic, Oslo, Norway – 150 from each site.^36^ The injury diagnosis was based on magnetic resonance imaging and anterior knee joint laxity measurement using the KT-1000 arthrometer (MEDmetric).^8^ Inclusion criteria were age between 13 and 60 years old and participation in pivoting sports (level 1 or 2) at least twice a week at the time of injury.^20,23^ We excluded participants with bilateral injuries, symptomatic concomitant injuries, or previous knee injuries. Among the 300 included participants, 24 had had a previous ACL reconstruction (ACLR) and came to the consultation with a graft rupture; they were excluded from the present study. Finally, 276 participants with a primary ACL injury were included at baseline.

After resolution of acute impairments at inclusion, all participants received 10 sessions of preoperative rehabilitation as described previously.^12^ Participants were educated about treatment alternatives, underwent functional testing, and then decided on their treatment strategy in a shared decision-making process together with their orthopaedic surgeon and physical therapist. ACLR was more likely recommended to patients experiencing dynamic knee instability after preoperative rehabilitation or planning to return to level 1 sports.^20,23,54^ The reason for pursuing rehabilitation alone was based mainly on achieving good knee function after rehabilitation. Delayed ACLR was performed for patients who experienced dynamic knee instability or if they changed their minds about the treatment choice.^54^ Further information about the treatment algorithm and rehabilitation program have been described in previous work.^19,20^

**Figure 1.**
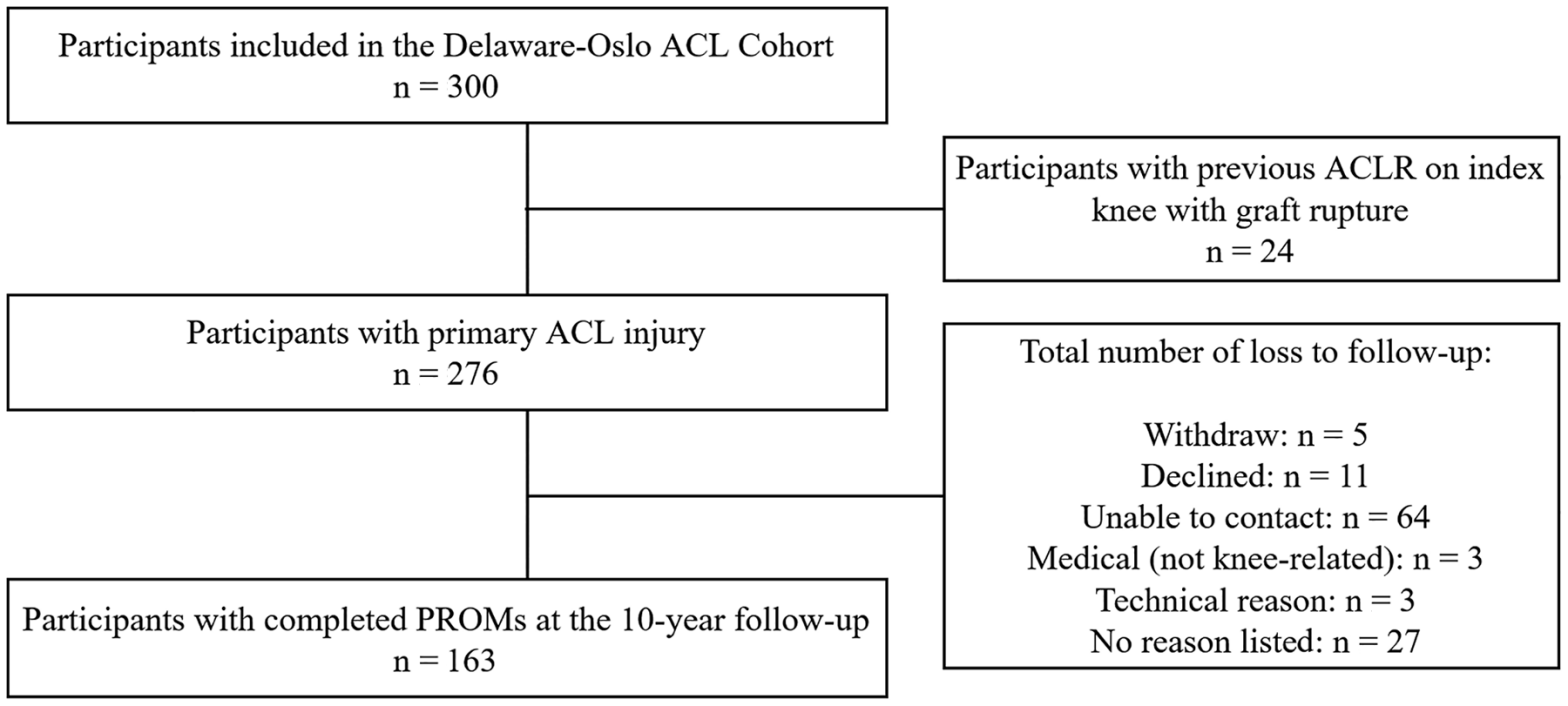
Study flowchart. ACL, anterior cruciate ligament; ACLR, anterior cruciate ligament reconstruction; PROMs, patient-reported outcome measures.

Ten years after completion of preoperative rehabilitation or after ACLR, 5 participants withdrew from the study, 11 declined to attend, 64 could not be contacted, 3 declined due to medical reasons, 3 had technical problems with the electronic questionnaire, and 27 had no reason listed. Finally, 163 (59%) participants were included in the analysis of this study (Figure 1). The rationale to include the whole cohort (including participants with different treatments) was based on previous findings from both the 5-year and 10-year follow-ups, which demonstrated that participants who followed rehabilitation alone had similar outcomes compared with participants treated with ACLR.^35,36,48^

### Data Collection

Questionnaires were sent to participants for the 10-year follow-up. The questionnaire included the IKDC-SKF, KOOS, PASS, and Treatment Failure anchor questions. The IKDC-SKF is scored from 0 (worst) to 100 (best).^26^ The IKDC-SKF has good test-retest reliability,^49^ construct validity,^1,27^ and responsiveness in participants after ACL injury.^27^ The KOOS comprises 5 separate subscales: pain, symptoms, activities of daily living (ADL), function in sport and recreation (Sports), and knee-related quality of life (QoL).^41^ All subscale scores were calculated using KOOS guidelines and scores ranged from 0 (worst/extreme problems) to 100 (best/no problems).^39^ The KOOS has good internal consistency, test-retest reliability, construct validity, and responsiveness in participants after ACL injuries and reconstruction.^6,15,28^ To identify PASS, we asked a first single-item anchor question^24^: “Considering your knee function, do you feel that your current state is satisfactory? With knee function, you should take into account all activities during your daily life, sport and recreational activities, your level of pain and other symptoms, and also your knee-related quality of life.” Answer options were dichotomized as “yes” or “no.” Participants answering “no” were asked a second anchor question related to Treatment Failure^24^: “Would you consider your current state as being so unsatisfactory that you think the treatment has failed?” Answer options were again dichotomized as “yes” or “no.” Similar PASS questions have good test-retest reliability in this patient population (κ = 0.78).^33^

### Statistical Methods

Individual characteristics at the 10-year follow-up were presented as means and standard deviations for continuous variables and percentage distribution for categorical variables. Normality was assessed using Shapiro-Wilk tests and Q-Q plots,^50^ and we considered data to be normally distributed and suitable for use with parametric tests. Group differences were assessed using Student *t* tests for continuous variables and with chi-square analysis of proportions or Fisher exact tests for categorical variables. Participants with missing data for the PASS question were excluded from our analysis; participants with missing data and participants with complete data were compared for sex, age, body mass index, months from injury to surgery (for ACLR only), and preoperative IKDC-SKF and KOOS scores.

Participants answering “yes” to the first PASS question were classified as having satisfactory symptom state. Those answering “no” were classified as having unsatisfactory symptom state. Participants answering “no” to the first question and then “yes” to the second question were classified as Treatment Failure.

Two modeling approaches were used to derive thresholds by anchoring IKDC-SKF and KOOS scores to the anchor questions. First, we calculated the commonly used ROC-derived thresholds using ROC analysis (no adjustment for proportion of reported answer choices or anchor question reliability).^45^ The optimal cutoff values were determined using the Youden index.^55^ Second, we calculated predictive modeling thresholds using adjusted predictive modeling,^43,44^ involving logistic regression using the dichotomous anchor answer as the dependent variable and the PROM scores as the independent variable. Bias occurs when the reported proportion of satisfactory symptom state differs from 50%,^43^ resulting in overestimated (or underestimated) thresholds if the proportion is > 50% (or < 50%). This effect is amplified by poor reliability of the anchor question. The item-score reliability of the anchor question was estimated by using confirmatory factor analysis (CFA).^10,18,44^ We adjusted the thresholds for the proportion of reported satisfactory symptom state and anchor question reliability using the following equation^44^: *predictive modeling thresholds* = *PASS_predictive_* - (0.08/*Rel_PASS_* - 0.5) ×*SD_score_*×*Cor*) ×*log-odds*(*PASS*). In this equation, Rel_PASS_ is the anchor question reliability, SD_score_ is the standard deviation of the PROM scores, Cor is the Pearson point-biserial correlation between the PROM scores and the anchor, and log-odds(PASS) is the natural logarithm of proportion having satisfactory symptom state divided by 1 minus the proportion having no satisfactory symptom state. Point-biserial correlation between the dichotomized anchors and the PROM scores were calculated as simple measures of anchor validity. We had no predefined correlation criterion due to inconsistency in literature. We used bootstrapping of 1000 random replications to obtain 95% CIs reported as 0.025 to 0.975 quantiles.

To assess criterion validity, we calculated the percentages of participants whose outcome scores exceeded these thresholds and were classified as PASS and compared them with the actual true PASS percentage based on the answer to the anchor question. In addition, we used a contingency table to calculate the sensitivity, specificity, positive predictive value, and negative predictive value of the predictive modeling thresholds and the ROC-derived thresholds for each PROM and subscale. Results are shown for the whole cohort, patients who underwent either ACLR or rehabilitation alone. In a subanalysis, we calculated thresholds for participants who underwent ACLR. All analyses were performed in R Version 4.2.3 (www.r-project.org).^38^

## Results

### Participants

A total of 116 participants underwent ACLR (92 underwent early ACLR ≤6 months after the 10-session rehabilitation program, 24 underwent delayed ACLR >6 months after the 10-session rehabilitation program), 46 followed rehabilitation alone, and 1 treatment status was unconfirmed (Table 1). The median (Q1-Q3) time from injury to surgery was 4.9 months (3.3-8.6 months); hamstring tendon autografts were used in 63 participants, patella tendon autografts were used in 32 participants, allografts were used in 19 participants, and the graft type remained unknown for 2 participants. Participants with missing data had higher body mass index (mean difference, 2.3 kg/m^2^) at inclusion compared with those with complete data (Supplemental Table S1).

**Table 1:** Descriptive Characteristics Overall at the 10-Year Follow-up After ACL Injury (N = 163)^*a*^

Descriptive Characteristics	Value^*b*^
Male sex	83 (51%)
Age, y	38.2 (9.6)
Body mass index, kg/m^2^	25.8 (4.0)
Years from ACL injury to follow-up	10.9 (0.8)
Treatment choice	
ACLR	116 (72%)
Rehabilitation alone	46 (28%)
PASS	127 (78%)
Treatment failure^*c*^	4 (2.5%)
IKDC-SKF (0-100)	87.0 (11.1)
KOOS Pain (0-100)	93.8 (7.8)
KOOS Symptoms (0-100)	90.3 (11.3)
KOOS ADL (0-100)	98.2 (4.1)
KOOS Sports (0-100)	86.4 (16.1)
KOOS QoL (0-100)	75.8 (19.4)

a ACLR, anterior cruciate ligament reconstruction; ADL, activities of daily living; IKDC-SKF, International Documentation Committee Subjective Knee Form; KOOS, Knee injury and Osteoarthritis Outcome Score; PASS, Patient Acceptable Symptom State; QoL, quality of life.

b n (%) or mean (SD).

c Of the 36 participants, 4 did not answer this question.

### PASS and No PASS, Based on the Anchor Questions

The percentages across the whole cohort were 78% (n = 127) for PASS and 22% (n = 36) for no PASS. Only 4 participants (2.5%) reported treatment failure, while another 4 participants (2.5%) did not answer the Treatment Failure question.

### Threshold Values for IKDC-SKF and KOOS Subscales

PASS thresholds were calculated with the predictive modeling and ROC analysis (Table 2). The 95% CIs indicated that predictive modeling thresholds were different from ROC analysis thresholds, with predictive modeling thresholds being lower by at least 10 points except for KOOS ADL. The point-biserial correlations between the dichotomized PASS anchor and the PROM scores were lowest (0.29) for KOOS Symptoms and highest (0.58) for KOOS QoL. We calculated the optimized CFA model fit and the estimated anchor question reliability for predictive modeling (Supplemental Table S2). Thresholds for participants who underwent ACLR were close to those from the whole cohort (results not presented). No thresholds were calculated for Treatment Failure due to the small number of participants reporting failed treatment (n = 4).

**Table 2:** PASS Thresholds Calculated With Predictive Modeling and ROC Analysis^*a*^

	Correlation Coefficient With PASS Anchor Question	Predictive Modeling PASS Thresholds	ROC-Derived PASS Thresholds
IKDC-SKF	0.56	76.2 (72.1-79.4)	86.8 (82.2-86.8)
KOOS Pain	0.41	85.4 (80.9-89.2)	95.8 (90.3-95.8)
KOOS Symptoms	0.29	76.5 (67.8-84.7)	94.6 (87.5-98.2)
KOOS ADL	0.31	93.8 (90.1-96.9)	99.3 (96.3-99.3)
KOOS Sports	0.45	71.6 (63.4-77.7)	82.5 (82.5-92.5)
KOOS QoL	0.58	59.0 (53.7-63.9)	71.9 (59.4-78.1)

a Correlation coefficients with the PASS question and PASS threshold values (95% CIs) calculated with predictive modeling and ROC for IKDC-SKF and KOOS subscales at the 10-year follow-up after ACL injury. ACL, anterior cruciate ligament; ADL, activities of daily living; IKDC-SKF, International Documentation Committee Subjective Knee Form; KOOS, Knee injury and Osteoarthritis Outcome Score; PASS, Patient Acceptable Symptom State; QoL, quality of life; ROC, receiver operating characteristic.

### Criterion Validity of the Thresholds

**Figure 2.**
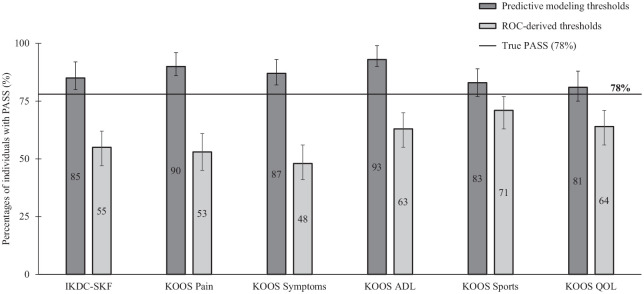
Percentages with 95% CIs of participants with IKDC-SKF and KOOS scores that were classified as PASS based on thresholds derived from predictive modeling (dark gray) or ROC (light gray). The horizontal line represents the true PASS percentage based on the anchor question (78%). ADL, activities of daily living; IKDC-SKF, International Documentation Committee Subjective Knee Form; KOOS, Knee injury and Osteoarthritis Outcome Score; PASS, Patient Acceptable Symptom State; QoL, quality of life; ROC, receiver operating characteristic.

The PASS thresholds derived from predictive modeling classified 81% to 93% of the participants as having PASS, whereas the ROC-derived PASS thresholds resulted in 48% to 71% (Figure 2). The difference in percentage (percentage points [pp]) to the true PASS percentage (78%) was smallest for predictive modeling thresholds in KOOS QoL (3 pp higher), KOOS Sports (5 pp higher), and IKDC-SKF (7 pp higher) (Figure 2). The ROC-derived thresholds were between 7 pp (KOOS Sports) and 30 pp (KOOS Symptoms) lower than the true PASS (Figure 2). Table 3 presents the false positives, false negatives, sensitivity, specificity, positive predictive value, and negative predictive value of the 2 methods of calculating PASS thresholds for each PROM and subscale. Of the participants who reported no PASS, predictive modeling thresholds correctly classified 73% to 86%, while ROC-derived thresholds correctly classified 40% to 61%. The predictive modeling thresholds for KOOS QoL had the highest combined positive and negative predictive values.

**Table 3:** Predictive Modeling and ROC-Derived PASS Thresholds^*a*^

	False Positives	False Negatives	Sensitivity (95% CI)	Specificity (95% CI)	PPV (95% CI)	NPV (95% CI)
Predictive Modeling PASS Thresholds						
IKDC-SKF	22	11	0.92 (0.05)	0.62 (0.12)	0.85 (0.06)	0.77 (0.12)
KOOS Pain	28	9	0.93 (0.04)	0.56 (0.12)	0.82 (0.06)	0.80 (0.12)
KOOS Symptoms	28	13	0.91 (0.05)	0.56 (0.12)	0.82 (0.06)	0.73 (0.12)
KOOS ADL	30	6	0.95 (0.04)	0.55 (0.12)	0.81 (0.06)	0.86 (0.11)
KOOS Sports	22	13	0.91 (0.05)	0.62 (0.12)	0.85 (0.06)	0.73 (0.12)
KOOS QoL	14	9	0.93 (0.04)	0.72 (0.12)	0.90 (0.05)	0.80 (0.12)
ROC-Derived PASS Thresholds						
IKDC-SKF	3	41	0.76 (0.06)	0.92 (0.09)	0.98 (0.02)	0.47 (0.11)
KOOS Pain	5	46	0.73 (0.07)	0.88 (0.10)	0.96 (0.03)	0.44 (0.11)
KOOS Symptoms	7	55	0.70 (0.07)	0.84 (0.11)	0.95 (0.04)	0.40 (0.10)
KOOS ADL	12	36	0.78 (0.06)	0.75 (0.12)	0.91 (0.05)	0.50 (0.12)
KOOS Sports	11	23	0.85 (0.06)	0.77 (0.12)	0.92 (0.05)	0.61 (0.12)
KOOS QoL	7	30	0.81 (0.06)	0.84 (0.11)	0.95 (0.04)	0.55 (0.12)

a False positives (above PASS thresholds), false negatives (below PASS thresholds), sensitivity, specificity, positive predictive value, and negative predictive value of PASS thresholds calculated with predictive modeling and receiver operating characteristic (ROC) for detecting true PASS at the 10-year follow-up after ACL injury. ADL, activities of daily living; IKDC-SKF, International Documentation Committee Subjective Knee Form; KOOS, Knee injury and Osteoarthritis Outcome Score; NPV, negative predictive value; PASS, Patient Acceptable Symptom State; PPV, positive predictive value; QoL, quality of life; ROC, receiver operating characteristic.

## Discussion

Our study revealed that predictive modeling PASS thresholds accurately reflected patient satisfaction, providing clinicians with a better understanding of group-based satisfactory outcomes at a 10-year follow-up after ACL injury. Predictive modeling yielded valid thresholds, with IKDC-SKF having a threshold of 76.2 points and KOOS subscales thresholds ranging from 59.0 to 93.8 points. Overall, 78% of participants reported PASS. While predictive modeling thresholds classified 81% to 93% of the participants as having satisfactory symptom state, ROC-derived thresholds classified more than 50% as unsatisfied. Among the thresholds identified, IKDC-SKF, KOOS Sports, and QoL resulted in the most accurate classification of participants with satisfactory symptom state.

### PASS Thresholds for IKDC-SKF and KOOS Subscales

To our knowledge, this is the first study to identify valid PASS thresholds for the IKDC-SKF and the KOOS subscales at a 10-year follow-up after ACL injury.^2,16,30^ Predictive modeling thresholds for IKDC-SKF and 4 KOOS subscales were considerably lower than the thresholds derived from ROC analysis, with a difference of >10 points. While no other study has calculated PASS thresholds for long-term outcomes after ACL injury,^30^ 2 studies have proposed thresholds for short-term IKDC-SKF and KOOS using the ROC method: the proposed thresholds were lower than our predictive modeling thresholds, by up to 9 points for KOOS QoL at 6 to 24 months post-ACLR and up to 19 points for KOOS Symptoms at 1 to 5 years post-ACLR.^3,33^ The difference was amplified when comparing both studies’ findings with our ROC-derived thresholds, with up to 22 and 38 points lower thresholds, respectively.^3,33^ Since thresholds are specific to the study population, the difference may be attributed to various factors of the included cohort (eg, demographics, treatment), especially the different follow-up time.^16^ We therefore recommend using the thresholds established in the present study in a population similar to the present study sample.

### Criterion Validity of the PASS Thresholds

Predictive modeling thresholds slightly overestimated the proportions of satisfactory outcomes; the difference in percentage to the true PASS percentage was only 3 pp for KOOS QoL, 5 pp for KOOS Sports, and 7 pp for IKDC-SKF. Importantly, ROC-derived thresholds substantially underestimated the true PASS percentage of 78%, notably in KOOS Symptoms (30 pp), KOOS Pain (25 pp), and IKDC-SKF (23 pp). This finding illustrates how thresholds with poor validity can skew the clinical interpretation of outcomes.

The good criterion validity of predictive modeling thresholds is attributed to the reduced bias caused by adjusting for the proportion of reported answer choices for PASS and anchor question reliability, which is particularly important when this proportion deviates from 50%.^43^ In contrast, ROC-derived thresholds performed poorly in classifying participants with unsatisfactory symptom state: up to 60% of participants classified as unsatisfied did in fact report true PASS, which is a substantial underestimation of satisfactory outcomes. We provided the thresholds from ROC analysis for those interested in comparison of methodologies but advise against applying them for clinical or research purposes.

### Applicability of PASS Thresholds

PASS is a unidimensional measure of patient satisfaction, whereas the IKDC-SKF and KOOS assess multiple dimensions of knee symptoms and function, making it challenging to determine a single, concise threshold that indicates patients’ satisfaction with their knee status. We found that predictive modeling PASS thresholds for the KOOS QoL, KOOS Sports, and IKDC-SKF resulted in percentages of participants with satisfactory symptom state that came closest to the true PASS percentage. Both KOOS subscales and the IKDC-SKF also had the strongest correlation with the PASS anchor. These findings suggest that, for KOOS, the Sports and QoL subscales capture the concerns most relevant to patients with an ACL injury.^21,25^ Therefore, and in line with previous recommendations from a systematic review of the psychometric properties of KOOS,^6^ clinicians should prioritize these subscales when determining symptom state in participants with ACL injuries. We also recommend incorporating IKDC-SKF for determining symptom state given its high accuracy and strong correlation with the PASS anchor.

PASS thresholds are probabilistic values applicable in a population similar to our study sample. The thresholds established in this study serve as group-based reference values for estimating satisfactory symptom state, notably for ACL registries or cohorts with 10-year follow-up. However, clinicians should be aware that individual thresholds might differ from group means, as patient perceptions about satisfactory symptom state vary. In such cases, using the confidence intervals from predictive modeling proves valuable. In addition, the PASS might be influenced by patient age and change in lifestyle due to age and expectations.^31^ Our findings have high external validity, including participants from different geographical regions (United States and Scandinavia) who were active in jumping, pivoting, or cutting sports preinjury and received treatment that aligned with current clinical recommendations.^13^

Previous studies that assessed group-based satisfactory symptom state at 10-year follow-up had to rely on ROC-derived thresholds that were calculated for a sample of patients who were between 1 and 5 years after ACLR.^4,17,22, 30,33,37,46,51^ While these thresholds may have limited validity for their given PROM and sample, they were also between 6 points higher to 19 points lower (KOOS Symptoms) when compared with our predictive modeling thresholds. For effective interpretation, we therefore recommend future studies use thresholds that are derived from predictive modeling and specific to patient characteristics, notably treatment and follow-up period.^16^

### Limitations

A limitation of this study is the loss to follow-up rate of 41%, which may introduce selection bias. Participants who did not return for the 10-year follow-up had higher baseline body mass index than those who did, possibly influencing the generalizability of the findings. Another shortcoming is that the ceiling effect of the PROM scores might underestimate the thresholds. The adjustment of the predictive modeling method requires using CFA to estimate the reliability of the anchor question. Model fit for some subscales was suboptimal due to low anchor question reliability.^43^ When the proportion of reported answer choices lies outside 30% and 70%, as in the present study population, some residual bias will remain depending on the reliability of the anchor question;^43^ this might lead to overestimated thresholds.

## Conclusion

This study provides PASS thresholds that accurately reflect patient satisfaction on IKDC-SKF and KOOS at the 10-year follow-up after ACL injury. These thresholds can help clinicians and policymakers to interpret long-term satisfactory symptom state at a group level, such as in national ACL registries, large cohorts, or clinical trials. Predictive modeling thresholds were valid for detecting satisfactory symptom state, while ROC-derived thresholds misclassified a substantial percentage of participants with satisfactory symptom state as unsatisfactory. When determining group-based symptom state after ACL injuries, clinicians should prioritize the predictive modeling thresholds for IKDC-SKF, KOOS Sports, and KOOS QoL.

## Supplemental Material

sj-pdf-1-ojs-10.1177_23259671241250025 – Supplemental material for Patient Acceptable Symptom State Thresholds for IKDC-SKF and KOOS at the 10-Year Follow-up After Anterior Cruciate Ligament Injury: A Study From the Delaware-Oslo ACL Cohort Supplemental material, sj-pdf-1-ojs-10.1177_23259671241250025 for Patient Acceptable Symptom State Thresholds for IKDC-SKF and KOOS at the 10-Year Follow-up After Anterior Cruciate Ligament Injury: A Study From the Delaware-Oslo ACL Cohort by Anouk P. Urhausen, Hege Grindem, Lina H. Ingelsrud, Ewa M. Roos, Karin Grävare Silbernagel, Lynn Snyder-Mackler and May Arna Risberg in Orthopaedic Journal of Sports Medicine

## References

[1] Anderson AF Irrgang JJ Kocher MS Mann BJ Harrast JJ , International Knee Documentation Committee. The International Knee Documentation Committee Subjective Knee Evaluation Form: normative data. Am J Sports Med. 2006;34(1):128-135. 16219941 10.1177/0363546505280214

[2] Beiene ZA Tanghe KK Kahlenberg CA McLawhorn AS MacLean CH Gausden EB. Defining a successful total knee arthroplasty: a systematic review of metrics of clinically important changes. Arthroplasty. 2023;5(1):25. 37198708 10.1186/s42836-023-00178-3 PMC10193600

[3] Beletsky A Naami E Lu Y , et al. The patient acceptable symptomatic state in primary anterior cruciate ligament reconstruction: predictors of achievement. Arthroscopy. 2021;37(2):600-605. 32911006 10.1016/j.arthro.2020.08.029

[4] Bergerson E Persson K Svantesson E , et al. Superior outcome of early ACL reconstruction versus initial non-reconstructive treatment with late crossover to surgery: a study from the Swedish National Knee Ligament Registry. Am J Sports Med. 2022;50(4):896-903. 35107352 10.1177/03635465211069995 PMC8980451

[5] Calvert M Blazeby J Altman DG , et al. Reporting of patient-reported outcomes in randomized trials: the CONSORT PRO extension. JAMA. 2013;309(8):814-822. 23443445 10.1001/jama.2013.879

[6] Collins NJ Prinsen CA Christensen R Bartels EM Terwee CB Roos EM. Knee Injury and Osteoarthritis Outcome Score (KOOS): systematic review and meta-analysis of measurement properties. Osteoarthritis Cartilage. 2016;24(8):1317-1329. 27012756 10.1016/j.joca.2016.03.010

[7] Cristiani R Mikkelsen C Edman G Forssblad M Engström B Stålman A. Age, gender, quadriceps strength and hop test performance are the most important factors affecting the achievement of a patient-acceptable symptom state after ACL reconstruction. Knee Surg Sports Traumatol Arthrosc. 2020;28(2):369-380. 31230125 10.1007/s00167-019-05576-2 PMC6994649

[8] Daniel DM Stone ML Sachs R Malcom L. Instrumented measurement of anterior knee laxity in patients with acute anterior cruciate ligament disruption. Am J Sports Med. 1985;13(6):401-407. 4073348 10.1177/036354658501300607

[9] Davis JC Bryan S. Patient reported outcome measures (PROMs) have arrived in sports and exercise medicine: why do they matter? Br J Sports Med. 2015;49(24):1545-1546. 25807155 10.1136/bjsports-2014-093707

[10] Devji T Carrasco-Labra A Qasim A , et al. Evaluating the credibility of anchor based estimates of minimal important differences for patient reported outcomes: instrument development and reliability study. BMJ. 2020;369:m1714. 32499297 10.1136/bmj.m1714 PMC7270853

[11] Dougados M. It’s good to feel better but it's better to feel good. J Rheumatol. 2005;32(1):1-2. 15630716

[12] Eitzen I Moksnes H Snyder-Mackler L Risberg MA. A progressive 5-week exercise therapy program leads to significant improvement in knee function early after anterior cruciate ligament injury. J Orthop Sports Phys Ther. 2010;40(11):705-721. 20710097 10.2519/jospt.2010.3345 PMC3158986

[13] Filbay SR Grindem H. Evidence-based recommendations for the management of anterior cruciate ligament (ACL) rupture. Best Pract Res Clin Rheumatol. 2019;33(1):33-47. 31431274 10.1016/j.berh.2019.01.018 PMC6723618

[14] Franceschini M Boffa A Pignotti E Andriolo L Zaffagnini S Filardo G. The minimal clinically important difference changes greatly based on the different calculation methods. Am J Sports Med. 2023;51(4):1067-1073. 36811558 10.1177/03635465231152484 PMC10026158

[15] Gagnier JJ Shen Y Huang H. Psychometric properties of patient-reported outcome measures for use in patients with anterior cruciate ligament injuries: a systematic review. JBJS Rev. 2018;6(4):e5. 10.2106/JBJS.RVW.17.00114 29634589

[16] Georgopoulos V Smith S McWilliams DF , et al. Harmonising knee pain patient-reported outcomes: a systematic literature review and meta-analysis of patient acceptable symptom state (PASS) and individual participant data (IPD). Osteoarthritis Cartilage. 2023;31(1):83-95. 36089231 10.1016/j.joca.2022.08.011

[17] Grassi A Macchiarola L Lucidi GA , et al. Ten-year survivorship, patient-reported outcome measures, and patient acceptable symptom state after over-the-top hamstring anterior cruciate ligament reconstruction with a lateral extra-articular reconstruction: analysis of 267 consecutive cases. Am J Sports Med. 2021;49(2):374-383. 33523751 10.1177/0363546520986875

[18] Griffiths P Terluin B Trigg A Schuller W Bjorner JB. A confirmatory factor analysis approach was found to accurately estimate the reliability of transition ratings. J Clin Epidemiol. 2022;141:36-45. 34464687 10.1016/j.jclinepi.2021.08.029

[19] Grindem H Eitzen I Engebretsen L Snyder-Mackler L Risberg MA. Nonsurgical or surgical treatment of ACL injuries: knee function, sports participation, and knee reinjury: the Delaware-Oslo ACL Cohort study. J Bone Joint Surg Am. 2014;96(15):1233-1241. 25100769 10.2106/JBJS.M.01054 PMC4116562

[20] Grindem H Wellsandt E Failla M Snyder-Mackler L Risberg MA. Anterior cruciate ligament injury - who succeeds without reconstructive surgery? The Delaware-Oslo ACL Cohort study. Orthop J Sports Med. 2018;6(5):2325967118774255. 10.1177/2325967118774255 PMC5968666 29854860

[21] Hambly K Griva K. IKDC or KOOS: which one captures symptoms and disabilities most important to patients who have undergone initial anterior cruciate ligament reconstruction? Am J Sports Med. 2010;38(7):1395-1404. 20351201 10.1177/0363546509359678

[22] Hamrin Senorski E Svantesson E Spindler KP , et al. Ten-year risk factors for inferior knee injury and osteoarthritis outcome score after anterior cruciate ligament reconstruction: a study of 874 patients from the Swedish National Knee Ligament Register. Am J Sports Med. 2018;46(12):2851-2858. 30102869 10.1177/0363546518788325

[23] Hefti F Muller W Jakob RP Staubli HU. Evaluation of knee ligament injuries with the IKDC form. Knee Surg Sports Traumatol Arthrosc. 1993;1(3-4):226-234. 8536037 10.1007/BF01560215

[24] Ingelsrud LH Granan LP Terwee CB Engebretsen L Roos EM. Proportion of patients reporting acceptable symptoms or treatment failure and their associated KOOS values at 6 to 24 months after anterior cruciate ligament reconstruction: a study from the Norwegian Knee Ligament Registry. Am J Sports Med. 2015;43(8):1902-1907. 25977523 10.1177/0363546515584041

[25] Ingelsrud LH Terwee CB Terluin B , et al. Meaningful change scores in the knee injury and osteoarthritis outcome score in patients undergoing anterior cruciate ligament reconstruction. Am J Sports Med. 2018;46(5):1120-1128. 29517924 10.1177/0363546518759543

[26] Irrgang JJ Anderson AF Boland AL , et al. Development and validation of the international knee documentation committee subjective knee form. Am J Sports Med. 2001;29(5):600-613. 11573919 10.1177/03635465010290051301

[27] Irrgang JJ Anderson AF Boland AL , et al. Responsiveness of the International Knee Documentation Committee subjective knee form. Am J Sports Med. 2006;34(10):1567-1573. 16870824 10.1177/0363546506288855

[28] Johnson JL Irrgang JJ Risberg MA Snyder-Mackler L. Comparing the responsiveness of the global rating scale with legacy knee outcome scores: a Delaware-Oslo Cohort study. Am J Sports Med. 2020;48(8):1953-1960. 32515989 10.1177/0363546520924817 PMC7448061

[29] Mabrouk A Nwachukwu B Pareek A , et al. MCID and PASS in knee surgeries. Theoretical aspects and clinical relevance references. Knee Surg Sports Traumatol Arthrosc. 2023;31(6):2060-2067. 36897384 10.1007/s00167-023-07359-2

[30] Macri EM Young JJ Ingelsrud LH , et al. Meaningful thresholds for patient-reported outcomes following interventions for anterior cruciate ligament tear or traumatic meniscus injury: a systematic review for the OPTIKNEE consensus. Br J Sports Med. 2022;56(24):1432-1444. 35973755 10.1136/bjsports-2022-105497

[31] Maksymowych WP Richardson R Mallon C van der Heijde D Boonen A. Evaluation and validation of the patient acceptable symptom state (PASS) in patients with ankylosing spondylitis. Arthritis Rheum. 2007;57(1):133-139. 17266072 10.1002/art.22469

[32] Mokkink LB Terwee CB Patrick DL , et al. The COSMIN study reached international consensus on taxonomy, terminology, and definitions of measurement properties for health-related patient-reported outcomes. J Clin Epidemiol. 2010;63(7):737-745. 20494804 10.1016/j.jclinepi.2010.02.006

[33] Muller B Yabroudi MA Lynch A , et al. Defining thresholds for the patient acceptable symptom state for the IKDC subjective knee form and KOOS for patients who underwent ACL reconstruction. Am J Sports Med. 2016;44(11):2820-2826. 27474383 10.1177/0363546516652888

[34] O’Toole RV Castillo RC Pollak AN MacKenzie EJ Bosse MJ ; LEAP Study Group. Determinants of patient satisfaction after severe lower-extremity injuries. J Bone Joint Surg Am. 2008;90(6):1206-1211. 18519312 10.2106/JBJS.G.00492 PMC2657303

[35] Pedersen M Grindem H Berg B , et al. Low rates of radiographic knee osteoarthritis 5 years after ACL reconstruction or rehabilitation alone: the Delaware-Oslo ACL Cohort study. Orthop J Sports Med. 2021;9(8):23259671211027530. 10.1177/23259671211027530 PMC8375355 34423060

[36] Pedersen M Grindem H Johnson JL , et al. Clinical, functional, and physical activity outcomes 5 years following the treatment algorithm of the Delaware-Oslo ACL Cohort study. J Bone Joint Surg Am. 2021;103(16):1473-1481. 33999877 10.2106/JBJS.20.01731 PMC8376754

[37] Persson K Bergerson E Svantesson E , et al. Greater proportion of patients report an acceptable symptom state after ACL reconstruction compared with non-surgical treatment: a 10-year follow-up from the Swedish National Knee Ligament Registry. Br J Sports Med. 2022;56(15):862-869. 35396203 10.1136/bjsports-2021-105115 PMC9304118

[38] R Core Team. R: A language and environment for statistical computing. Accessed October 2022. https://www.r-project.org

[39] Roos EM . KOOS. Accessed September 2023. https://eprovide.mapi-trust.org/instruments/knee-injury-and-osteoarthritis-outcome-score

[40] Roos EM Boyle E Frobell RB Lohmander LS Ingelsrud LH. It is good to feel better, but better to feel good: whether a patient finds treatment “successful” or not depends on the questions researchers ask. Br J Sports Med. 2019;53(23):1474-1478. 31072841 10.1136/bjsports-2018-100260

[41] Roos EM Lohmander LS. The Knee injury and Osteoarthritis Outcome Score (KOOS): from joint injury to osteoarthritis. Health Qual Life Outcomes. 2003;1:64. 14613558 10.1186/1477-7525-1-64 PMC280702

[42] Roos EM Roos HP Lohmander LS Ekdahl C Beynnon BD. Knee Injury and Osteoarthritis Outcome Score (KOOS) - development of a self-administered outcome measure. J Orthop Sports Phys Ther. 1998;28(2):88-96. 9699158 10.2519/jospt.1998.28.2.88

[43] Terluin B Eekhout I Terwee CB. Improved adjusted minimal important change took reliability of transition ratings into account. J Clin Epidemiol. 2022;148:48-53. 35436522 10.1016/j.jclinepi.2022.04.018

[44] Terluin B Eekhout I Terwee CB. The anchor-based minimal important change, based on receiver operating characteristic analysis or predictive modeling, may need to be adjusted for the proportion of improved patients. J Clin Epidemiol. 2017;83:90-100. 28093262 10.1016/j.jclinepi.2016.12.015

[45] Terluin B Eekhout I Terwee CB de Vet HC. Minimal important change (MIC) based on a predictive modeling approach was more precise than MIC based on ROC analysis. J Clin Epidemiol. 2015;68(12):1388-1396. 25913670 10.1016/j.jclinepi.2015.03.015

[46] Thorolfsson B Lundgren M Snaebjornsson T , et al. Lower rate of acceptable knee function in adolescents compared with young adults five years after ACL reconstruction: results from the Swedish National Knee Ligament Register. BMC Musculoskelet Disord. 2022;23(1):793. 35982445 10.1186/s12891-022-05727-6 PMC9389739

[47] Tubach F Ravaud P Baron G , et al. Evaluation of clinically relevant states in patient reported outcomes in knee and hip osteoarthritis: the patient acceptable symptom state. Ann Rheum Dis. 2005;64(1):34-37. 15130902 10.1136/ard.2004.023028 PMC1755171

[48] Urhausen AP Pedersen M Grindem H et al. Good 10-Year Outcomes Following The Treatment Algorithm Of The Delaware-Oslo ACL Cohort: Knee Osteoarthritis, Symptoms And Function. Osteoarthritis Cart. 2023;31(Supp.1):S48-S49.

[49] van Meer BL Meuffels DE Vissers MM , et al. Knee injury and Osteoarthritis Outcome Score or International Knee Documentation Committee subjective knee form: which questionnaire is most useful to monitor patients with an anterior cruciate ligament rupture in the short term? Arthroscopy. 2013;29(4):701-715. 23402944 10.1016/j.arthro.2012.12.015

[50] Vetter TR. Fundamentals of research data and variables: the devil is in the details. Anesth Analg. 2017;125(4):1375-1380. 28787341 10.1213/ANE.0000000000002370

[51] Wang K Eftang CN Ulstein S Aroen A Jakobsen RB. Concomitant full-thickness cartilage lesions do not affect patient-reported outcomes at minimum 10-year follow-up after ACL reconstruction. Knee Surg Sports Traumatol Arthrosc. 2022;30(5):1836-1845. 34626228 10.1007/s00167-021-06757-8 PMC8501353

[52] Watt FE Corp N Kingsbury SR , et al. Towards prevention of post-traumatic osteoarthritis: report from an international expert working group on considerations for the design and conduct of interventional studies following acute knee injury. Osteoarthritis Cartilage. 2019;27(1):23-33. 30125638 10.1016/j.joca.2018.08.001 PMC6323612

[53] Whittaker JL Culvenor AG Juhl CB , et al. OPTIKNEE 2022: consensus recommendations to optimise knee health after traumatic knee injury to prevent osteoarthritis. Br J Sports Med. 2022;56(24):1393-1405. 36379676 10.1136/bjsports-2022-106299

[54] Williams GN Chmielewski T Rudolph K Buchanan TS Snyder-Mackler L. Dynamic knee stability: current theory and implications for clinicians and scientists. J Orthop Sports Phys Ther. 2001;31(10):546-566. 11665743 10.2519/jospt.2001.31.10.546

[55] Youden WJ. Index for rating diagnostic tests. Cancer. 1950;3(1):32-35. 15405679 10.1002/1097-0142(1950)3:1<32::aid-cncr2820030106>3.0.co;2-3

