# Supplementary material for: Patient Acceptable Symptom State Thresholds for IKDC-SKF and KOOS at the 10-Year Follow-up After Anterior Cruciate Ligament Injury: A Study From the Delaware-Oslo ACL Cohort
Source: Orthop J Sports Med. 2024 May 29;12(5):23259671241250025. doi: 10.1177/23259671241250025 (PMC11143835; doi:10.1177/23259671241250025)
Supplement: sj-pdf-1-ojs-10.1177_23259671241250025 – Supplemental material for Patient Acceptable Symptom State Thresholds for IKDC-SKF and KOOS at the 10-Year Follow-up After Anterior Cruciate Ligament Injury: A Study From the Delaware-Oslo ACL Cohort [file sj-pdf-1-ojs-10.1177_23259671241250025.pdf]

Supplemental Table S1. Comparison of descriptive characteristics at baseline between completers and noncompleters of the patient-reported outcomes at the 10-year follow-up after ACL injury

	**Completers**, N = 163[1]	**Noncompleters**, N = 113[1]
Inclusion site Oslo	118 (72%)	24 (21%)
Sex	83 (51%)	65 (58%)
Age	27.4 (9.7)	25.1 (9.8)
Body mass index (kg/m^2)	23.7 (3.1)	26.0 (4.8)
Cartilage injury[§]	18 (11%)	4 (3.5%)
Medial meniscus injury[§]	41 (25%)	23 (20%)
Lateral meniscus injury[§]	31 (19%)	17 (15%)
Meniscus surgery at time of ACLR[£]	47 (29%)	36 (32%)
IKDC-SKF (0-100)	79.4 (11.6)	77.5 (13.6)
KOOS Pain (0-100)	89.1 (9.1)	89.9 (9.8)
KOOS Symptoms (0-100)	82.7 (12.9)	87.4 (9.3)
KOOS ADL (0-100)	97.0 (4.1)	97.3 (4.7)
KOOS Sports (0-100)	76.3 (15.9)	79.3 (16.3)
KOOS QOL (0-100)	59.2 (17.1)	58.7 (22.7)

[1]n (%) or mean (SD)

[§]Number of patients diagnosed with the injury with use of MRI at the time of inclusion.
[£]Only applicable for individuals who underwent ACLR.

ADL, activities of daily living; IKDC-SKF, International Documentation Committee Subjective Knee Form; KOOS, Knee injury and Osteoarthritis Outcome Score; PASS, patient acceptable symptom state; QOL, quality of life; ROC, receiving operating characteristic

Supplemental Table S2. Parameters of the confirmatory factor analysis model fit and estimated anchor question reliability for the IKDC-SKF and all KOOS subscales for all included individuals (n=163) at the 10-year follow-up after ACL injury

	**IKDC-SKF**	**KOOS Pain**	**KOOS Symptoms**	**KOOS ADL**	**KOOS Sports**	**KOOS QOL**
**Model included correlations**	2 ~~ 3<br>1 ~~ 2<br>9g ~~ 9h<br>9h ~~ 9i	P7 ~~ P8	S2 ~~ S3<br>S4 ~~ S5<br>S6 ~~ S7	A9 ~~ A10<br>A5 ~~ A11<br>A5 ~~ A9	SP2 ~~ SP3	/
CFI-scaled	0.921	1.000	1.000	0.985	0.988	0.991
TLI-scaled	0.946	1.002	1.011	0.989	0.984	0.989
RMSEA-scaled	0.141	0.000	0.000	0.054	0.106	0.103
RMSEA CI low	0.127	0.000	0.000	0.029	0.037	0.000
RMSEA CI high	0.155	0.064	0.054	0.075	0.179	0.197
RMSEA p-value	0.000	0.870	0.940	0.369	0.080	0.126
SRMR	0.157	0.071	0.050	0.119	0.061	0.038
Anchor question reliability	0.549	0.353	0.232	0.266	0.452	0.670

ADL, activities of daily living; CFI (comparative fit index); IKDC-SKF, International Documentation Committee Subjective Knee Form; KOOS, Knee injury and Osteoarthritis Outcome Score; QOL, quality of life; RMSEA (root mean square error of approximation); SRMR (standardized root mean squared residual); TLI (Tucker–Lewis index)
